# A diverse gut virome in natural populations of *Drosophila melanogaster*

**DOI:** 10.1186/s42523-026-00585-2

**Published:** 2026-06-04

**Authors:** Mina Hojat Ansari, Fabian Staubach, Nurper Alacatli, Darren J. Obbard

**Affiliations:** 1 Department of Evolution and Ecology, University of Freiburg, Freiburg, Germany; 2 Bioinformatics Group, Department of Computer Science, University of Freiburg, Freiburg, Germany; 3 Institute of Ecology and Evolution, University of Edinburgh, Edinburgh, EH9 3FL UK

**Keywords:** Gut microbiome, DNA virus, Phage, Drosophila

## Abstract

**Background:**

*Drosophila melanogaster* is not only one of the most important models of antiviral immunity in invertebrates, but is also a powerful model for research of the gut microbiome. Although recent studies have continued to improve our knowledge of the fly gut microbiota, the viral component of the microbiome has remained unexplored.

**Results:**

Here we explore the viral component of the *Drosophila melanogaster* gut microbiome using deep metagenomic DNA sequencing. We recovered 3040 non-redundant viral contigs, most of which were bacteriophage-associated sequences, resulting in 167 viral Metagenome-Assembled Genomes. Many of these sequences showed limited similarity to reference viruses and included bacteriophages related to tailed double-strand DNA phage lineages, with putative links to major gut-associated bacteria of *D. melanogaster*, including *Lactobacillus*, *Acetobacter*, and *Gluconobacter*. Our functional annotation and discovery of auxiliary metabolic genes suggested that these bacteriophages encode putative functional potential related to microbial metabolism and genetic information processing. We also identified evidence of known fly pathogens Drosophila Kallithea nudivirus, Vesanto bidna-like virus, and Drosophila Linvill Road densovirus, some of which were common in our studied populations.

**Conclusions:**

Our findings reveal a complex and diverse phage community in the *D. melanogaster* gut microbiome, paving the way to study host-phage related research in the natural microbial communities.

**Supplementary Information:**

The online version contains supplementary material available at 10.1186/s42523-026-00585-2.

## Background

The study of viruses, including bacteriophage (phages; bacteria and archaea-infecting viruses) has recently garnered increasing interest in scientific communities due to the advancement of metagenomic techniques that facilitate the identification of viral genomes. Bacteriophages have been found in nearly all investigated ecosystems, including animal-associated microbiomes, and are recognized as the most abundant and diverse biological entities on Earth [1]. They play an important role in microbial communities by affecting microbial diversity and metabolism [2, 3], and mediating phage-borne antibiotic resistance within hosts [4].

Such studies have also revealed the presence of auxiliary metabolic genes (AMGs) in bacteriophages [5]. These genes encode proteins that can regulate metabolic processes within host cells. In addition, bacteriophages have been found to regulate transcription and translation [6], and influence the decision to lyse or enter a lysogenic state [7], further underscoring the importance of characterizing bacteriophages and their interactions with their hosts.

In humans, it has been demonstrated that bacteriophages are mechanistically involved in human health and disease [8]. However, information about phages as an important component of the microbiome infecting most species is still lacking. This is true even for the well-studied model organism *Drosophila*, despite it being a powerful model for gut microbiome research [9, 10] and for the study of evolution and ecology of host-microbe interactions [11, 12]. The fruit fly *D.* *melanogaster* is a model organism that naturally interacts with a variety of microorganisms due to its feeding, mating, and ovipositing on fermenting and rotten fruits. *Drosophila melanogaster* has not only been established as a powerful model for gut microbiome research [9, 10], but is also well known as an important model for invertebrate antiviral immunity [13, 14]. However, most studies aimed at describing viruses in *Drosophila* have focused on the detection of RNA viruses of the host [15, 16], and studies on the diversity of DNA viruses have exclusively described eukaryotic DNA viruses [17]. Thus, almost nothing is known about the diversity or function of prokaryotic viruses, particularly bacteriophages, associated with *Drosophila*. Because bacteriophages can shape bacterial community structure, metabolic potential, and evolutionary dynamics, the characterization of phage-bacteria interactions is essential for understanding microbiome-mediated effects on host biology. Here we aim to provide an initial characterization of the prokaryotic viral microbiome associated with *D. melanogaster* by applying metagenomic sequencing to screen the guts of wild-caught flies for viruses. While previous studies in Drosophila have focused primarily on RNA viruses and eukaryotic DNA viruses, this study extends existing work by characterizing bacteriophage diversity and potential phage-bacteria interactions within an animal-associated gut microbiome.

## Results

### The metagenomic virome of *D. melanogaster* gut

We processed 851 metagenomes generated from *D. melanogaster* gut samples, resulting in nearly 38 billion high quality reads (ranging 1 to 90 million per sample). After mapping to the host genome, we retained 7.7 billion non-fly reads (ranging 23,436 to 73 million per sample) for downstream analysis.

Viral sequences were identified using VIBRANT [18], VirSorter2 [19] and DeepVirfinder [20] (Supplementary Table S1). After filtering and dereplication, we identified 3040 non-redundant viral contigs (Supplementary Table S2). These contigs varied widely in length, including 940 contigs shorter than 3 kb and 1,145 contigs longer than 5 kb, reflecting the fragmented nature of metagenomic assemblies.

Additional validation using Cenote-Taker 2 identified terminal repeat features in a subset of contigs, including 32 contigs with direct terminal repeats (DTRs) and 16 with inverted terminal repeats (ITRs), supporting the presence of complete or near-complete viral genomes (Supplementary Table S2).

To further assess sequence quality, we applied CheckV and accordingly most of the contigs were classified as low-quality genome fragments (< 50% complete), with 67 medium-quality (50–90% complete) and 37 high-quality (> 90% complete) genomes, including 20 predicted complete genomes (Supplementary Fig. S1). In addition, 115 contigs were classified as not-determined. These contigs are unlikely to represent false positives, as they were retained only when supported by all three detection tools at a high-confidence threshold (≥ 0.9). Instead, they likely represent short or highly divergent sequences relative to current reference databases.

Based on these metrics, we defined a curated high-confidence subset of viral contigs for downstream biological interpretation. In this study, this subset primarily includes longer contigs (> 5 kb) and sequences supported by multiple detection approaches, which provide more reliable signals for ecological and functional inference. The full catalog is retained to document overall viral diversity. Unless explicitly stated otherwise, biological interpretations regarding ecology, lifestyle, host association, and functional potential are restricted to this high-confidence subset.

Next, we used VIBRANT to also predict viral lifestyle. At the catalog level, we found that 96.7% of them (2941 viral contigs) were predicted to have a lytic lifestyle, while only 3.3% represented a temperate lifestyle (Supplementary Table S2). When restricting the analysis to the high-confidence subset, a similar pattern was observed, with the majority of contigs predicted to be lytic (1,084), indicating that this trend is not driven solely by low-quality or fragmented sequences. However, because lifecycle predictions are inherently more reliable for longer and higher-quality viral genomes, all biological interpretation focuses on the defined high-confidence subset of viral contigs. Accordingly, the apparent dominance of a lytic lifestyle should be interpreted cautiously and primarily as a descriptive pattern rather than a strong biological conclusion for the full dataset.

### Protein classification and taxonomy of the virus contigs from the *D. melanogaster* gut

**Fig. 1:**
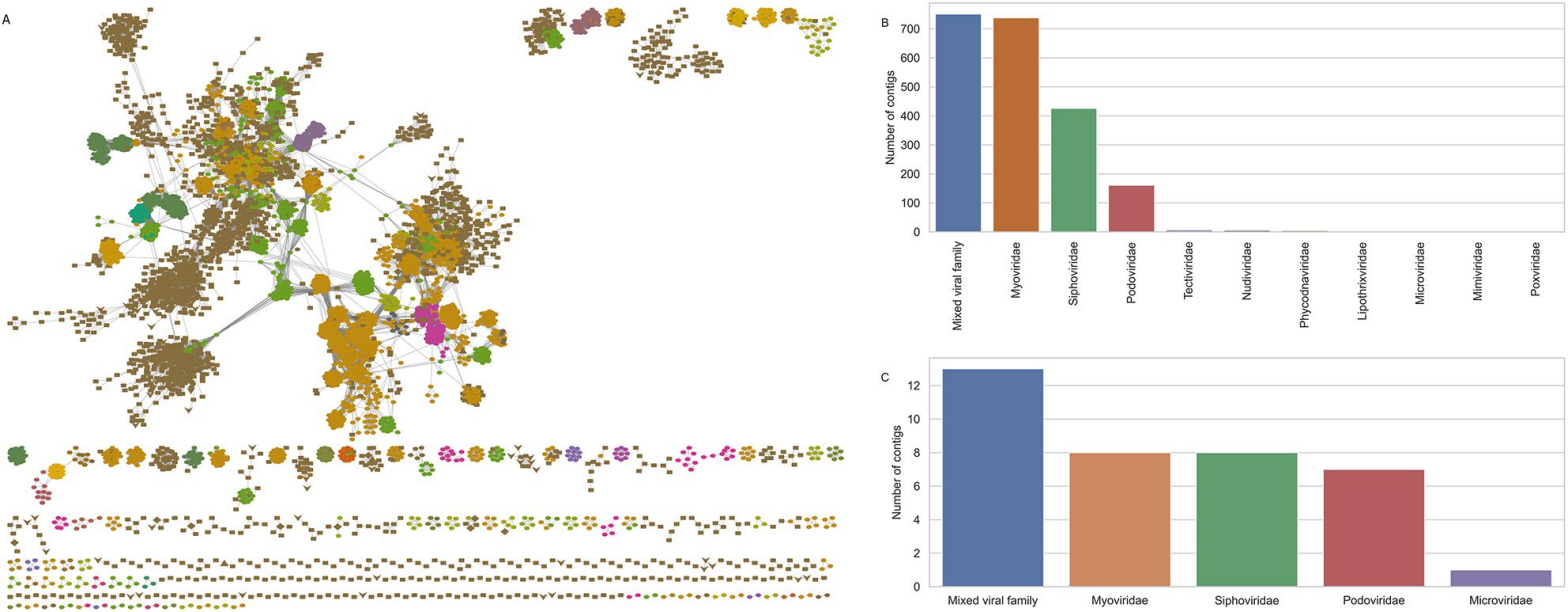
Taxonomy assignment and protein network clustering of viral sequences. **A**: Protein clustering network of the recovered viral sequences from *D. melanogaster* (dark brown) compared to previously described viruses from RefSeq (other colors). The most common viral families are highlighted with orange (*Siphoviridae*-like), green (*Myoviridae*-like), and olive (*Podoviridae*-like) colors. **B**: Taxonomy of viral sequences at family level, determined by VPF class. **C**: Viral families associated with high-quality viral sequences

We evaluated the degree to which the newly recovered *D. melanogaster* gut-associated viruses were represented in available public databases (Fig. 1). Using gene-sharing networks analysis (vConTACT2), we determined that of 3040 non-redundant contigs, 716 were singletons, 741 were outliers, 294 showed overlap with multiple established clusters (i.e. cannot be unambiguously assigned), and 1289 were clustered either with RefSeq sequences or among themselves (Supplementary Table S3 and Supplementary Fig. S2A).

Here, vConTACT2 analysis is used to describe gene-sharing relationships and the representation of viral sequences in current reference databases, rather than to provide definitive taxonomic classification. Accordingly, the 1289 clustered contigs formed 469 clusters, of which 434 clusters did not include any sequences from the RefSeq database (Fig. 1A and Supplementary Table S3). The remaining 35 clusters, representing 67 contigs, were associated with *Siphoviridae*-like, *Myoviridae*-like, and *Podoviridae*-like lineages based on gene-sharing network similarity (Supplementary Fig. S2B). Furthermore, members of *Schitoviridae*, *Herelleviridae*, and *Autographiviridae* were present, although they comprised only a small number of contigs (Fig. 1A and Supplementary Fig. S2B).

At the catalog level, the protein similarity network represented the newly identified phage sequences as broadly dispersed across the known viral diversity (Fig. 1A), while 25% of the recovered sequences remained unclassified. This pattern, together with the large number of singletons and small disconnected clusters observed in the network (Fig. 1A), indicates that a substantial fraction of the recovered viral diversity is not represented in current reference databases. However, clustering success and taxonomic assignment were strongly influenced by contig length and completeness. Higher quality contigs (> 50% complete) more frequently clustered with previously described viruses, with approximately 30% assigned to known phages (Supplementary Fig. S3). Similarly, assignment success was higher among larger contigs (> 5 kb), where 53 contigs (4.6%) were assigned to network-based clusters, compared with only 14 (~ 1%) of shorter contigs (Supplementary Figs. S4), indicating reduced classification power for shorter and more fragmented contigs. Importantly, the distribution of larger contigs across network-based lineages was broadly consistent with that observed for the full dataset (Fig. 1 and Supplementary Fig. S2 versus Supplementary Fig. S4).

To further characterize taxonomic signals, we analyzed viral protein families (VPF) compositions across contigs (Supplementary Table S4). Across the full dataset, VPF profiles indicated associations with major bacteriophage groups, including *Siphoviridae*-like, *Myoviridae*-like, and *Podoviridae*-like lineages, with lower representation of *Tectiviridae* and *Microviridae* (ssDNA viruses). We also identified VPFs from archaeal virus families (*Lipothrixviridae*, *Sphaerolipoviridae*, *Rudiviridae*, and *Turriviridae*), NCLDV, and *Nudiviridae*, a family of DNA viruses known to infect eukaryotes, including *Drosophila* (Fig. 1B and Supplementary Table S4). These patterns are consistent with a bacteriophage-dominated virome associated with the bacterial gut microbiota.

Consistent with this pattern, contigs longer than 5 kb showed similar VPF associations (Fig. 1B), with most assigned to *Myoviridae*-like, *Siphoviridae*-like, and *Podoviridae*-like lineages. In particular, among the 37 high-quality (> 90% completeness) genomes, VPF profiles (Fig. 1C) indicated affiliation with bacteriophage-associated lineages, including *Myoviridae*-like, *Siphoviridae*-like, *Podoviridae*-like, and *Microviridae*. Notably, 27 (73%) of these high-quality genomes did not cluster with known reference phages (Supplementary Fig. S3), suggesting that related viral groups remain underrepresented in current reference databases. Because these assignments are derived from available reference databases, including RefSeq v211, and because bacteriophage taxonomy and reference databases remain incomplete and under revision, these results should be interpreted as database-derived similarity patterns rather than definitive formal taxonomic assignments. Therefore, contigs lacking reference clusters are best interpreted as underrepresented in current databases rather than as precise estimates of novel taxa.

### Host assignment of bacteriophage contigs

Host assignments reported here represent putative computational predictions rather than experimentally validated host-phage relationships. To predict the hosts of the recovered phage sequences, we took advantage of the VPFs as well as CRISPR-spacer sequences.

Using the VPF approaches with a membership ratio and a confidence score cutoff of 0.6 and 0.8 respectively, 650 contigs could be linked to a host domain, of which almost all were assigned to bacterial host (Supplementary Table S5). Further investigation showed that 267 of these phage contigs were linked to 15 host families and 156 of them could be related to 20 host genera. All identified potential hosts reflect either the gut or environmental bacteria (Supplementary Table S5). The most frequently represented genera included *Pseudomonas*, *Lactococcus*, *Staphylococcus*, *Escherichia*, and *Lactobacillus* (Supplementary Table S5). Notably, 276 of the contigs contributing to these host assignments were substantially longer than 5 kb, which supports the broader host-association pattern.

On the other hand, the CRISPR-spacer approach, which was used to predict bacterial hosts of determined phages, linked 488 bacteriophage contigs to 61 unique bacterial genera. Of these 488 contigs, most of them (> 300 contigs) hit against bacteria commonly associated with the *Drosophila* gut, including *Lactobacillus*, *Gluconobacter*, *Acetobacter*, *Acinetobacter*, *Pseudomonas*, *Serratia*, *Corynebacterium*, *Klebsiella*, and *Komagataeibacter* (Supplementary Table S5).

Comparing the result from VPF and CRISPR-spacer approaches showed 29 contigs that hit the host at genus-level shared between two approaches, but only 69% of them (20 contigs) hit the same bacterial genus using both approaches (Supplementary Table S5). Further investigation showed that the VPF tools also predicted the presence of similar genus as CRISPR-spacer for these nine contigs, but were excluded due to our inclusion threshold (including only VPFs with a membership ratio and confidence score of ≥ 0.6 and ≥ 0.8, respectively).

Overall, both approaches indicate that the recovered bacteriophage contigs are predominantly associated with bacterial taxa characteristic of the *Drosophila* gut and its food-associated environment. Although individual host assignments remain computational predictions and should be interpreted cautiously, particularly for shorter contigs, the consistency between VPF-based prediction and CRISPR-spacer matching, together with similar signals observed among larger contigs, supports the broader host-association pattern in this dataset.

### Functional potential of *D. melanogaster* associated phage contigs from gut

Functional annotations are reported for the full catalog, however, interpretation of functional potential is more reliable for the high-confidence subset of contigs. Annotations from KEGG, Pfam, and VOG databases were used to gain insight into the functional potential encoded by the recovered contigs. Based on the available databases, 1549 contigs from the full catalog were assigned a function based on KEGG, representing 419 unique KEGG orthologs (KOs) (Supplementary Table S6). However, because short and fragmented contigs may provide less reliable genomic context, the main functional interpretation was focused on contigs longer than 5 kb. Within this subset, 806 contigs were assigned KEGG annotations, representing 395 unique KOs (Supplementary Table S6).

Consistent with the full dataset, among contigs longer than 5 kb, the majority of phage-associated functional genes were annotated with metabolism-related functions, including nucleotide, amino acid, lipid, energy and carbohydrate metabolism (Supplementary Table S6). Furthermore, functional genes associated with DNA repair, replication, and recombination, and transcription also represented a large proportion of the annotations (Supplementary Table S6). These functional annotations should be interpreted as putative genetic potential rather than evidence of active metabolic processes.

Across the full dataset, we identified 187 AMG annotations distributed across 148 phage contigs and assigned to 42 unique KOs (Supplementary Table S6). However, when focusing on contigs longer than 5 kb, 143 AMG annotations were retained across 108 phage contigs and assigned to 38 unique KOs. Among these contigs larger than 5 kb, the most abundant AMGs, in terms of number of contigs involved, included dcm, queE, folA/DHFR, folE/GCH1, mec, and NAMPT. These genes encode, respectively, DNA cytosine-5 methyltransferase (Dcm), the 7-cyano-7-deazaguanine synthase, dihydrofolate reductase, GTP cyclohydrolase IA, the [CysO sulfur carrier protein] -S-L-cysteine hydrolase, and nicotinamide phosphoribosyl transferase (Supplementary Table S6). In other words, the AMGs detected among *D. melanogaster* gut phage contigs tend to encode products mainly for amino acid, cofactor and vitamin metabolism and folding, sorting and degradation. As a conservative quality check, we also evaluated contigs longer than 5 kb that were classified by CheckV as at least medium quality. Within this subset, 28 AMG annotations were detected across 17 contigs, corresponding to 17 unique KOs (Supplementary Table S6). This lower number reflects the effect of stricter quality filtering rather than a contradiction with the full-catalog AMG count.

**Fig. 2:**
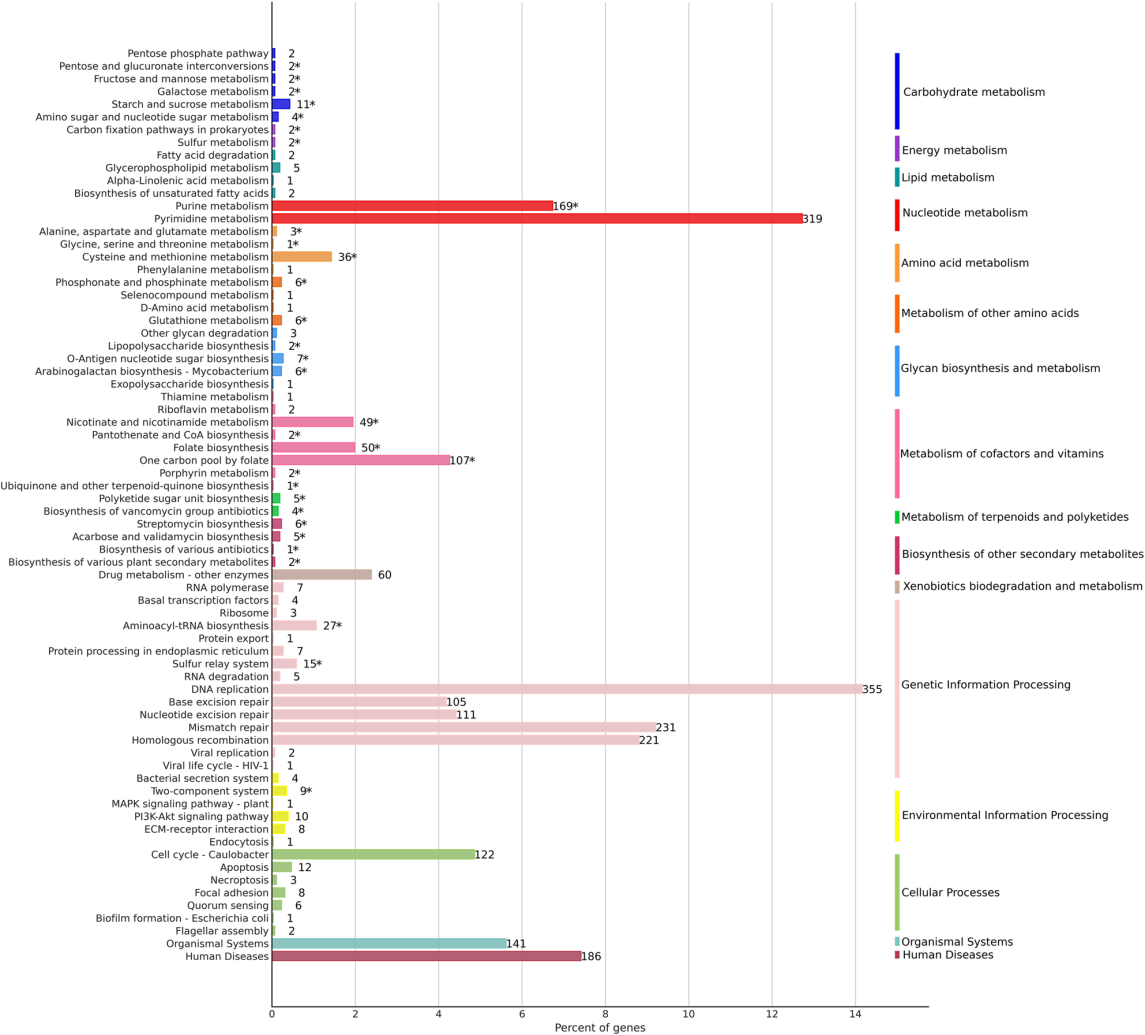
Bar plot illustrates the functional annotation results based on the KEGG pathways represented in the recovered viral sequences

To further explore the functional potential of the identified contigs, we projected KO accessions onto the KEGG pathways as a descriptive overview of the viral catalog (Fig. 2), while focusing interpretation on categories also represented among contigs longer than 5 kb. In some cases, the retrieved pathways represent functions that could directly affect bacteria, such as involvement in biofilm formation and bacterial secretion systems. In general, the retrieved pathways reflect a wide range of metabolic functions, including carbohydrate, energy, lipid, nucleotide, and amino acid metabolism. Furthermore, pathways related to glycan biosynthesis and cofactors, vitamins, xenobiotic degradation, and a limited number of terpenoid/polyketide-related functions were presented (Fig. 2). The identified genes in *D. melanogaster* gut phages are also involved in folding, sorting, degradation, transcription, and translation, as well as in replication and repair. KEGG pathway summaries are descriptive and were not subjected to formal hypothesis testing; therefore, no correction for multiple testing was applied.

### Genome binning of phage contigs from metagenomic data from *D. melanogaster*

**Fig. 3:**
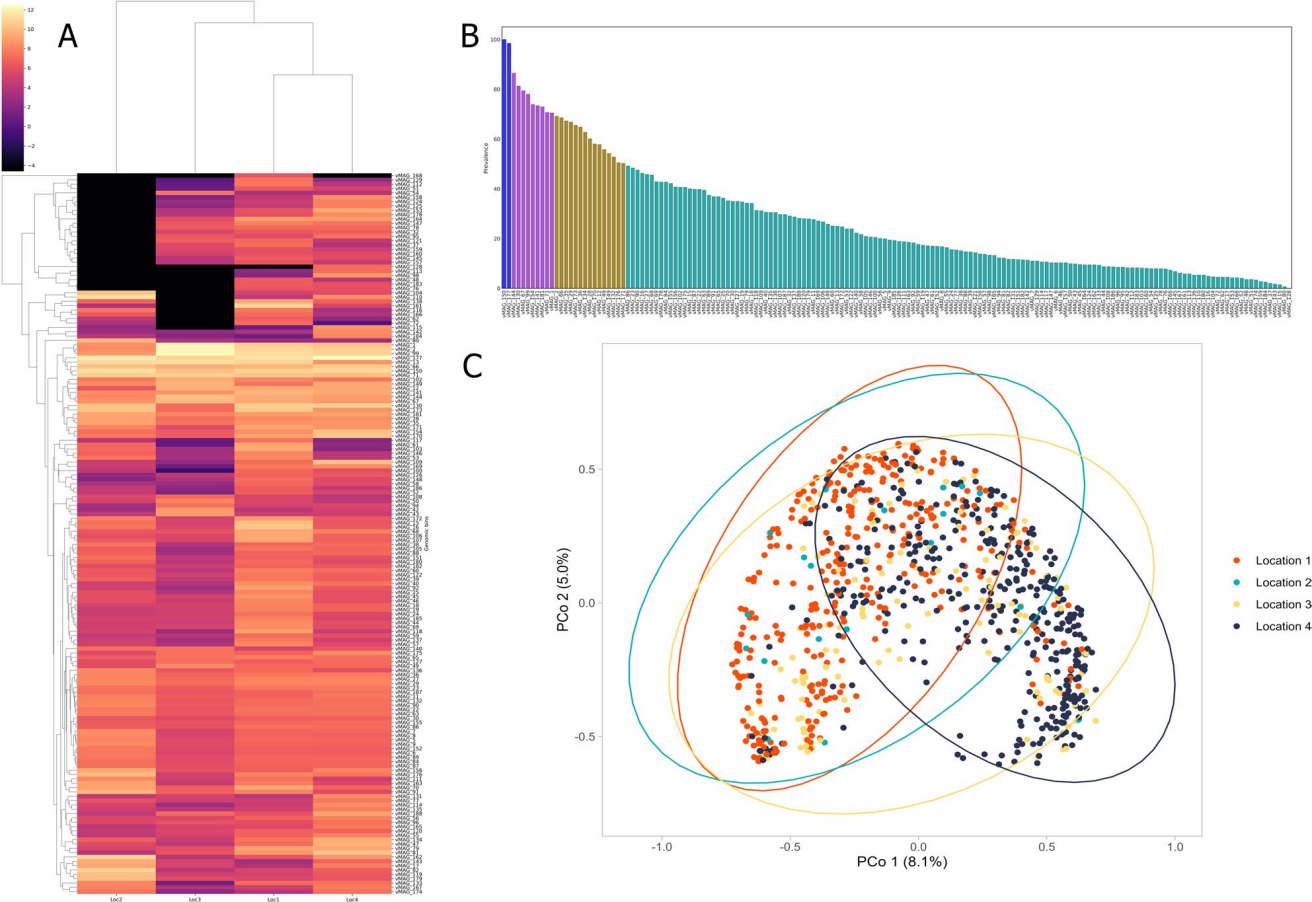
Characterization of the viral metagenome assembled genome (vMAG) recovered from *D. melanogaster* gut microbiome. **A**: Relative abundance and distribution of 167 recovered vMAGs from four sampling locations (Loc). **B**: The prevalence for each vMAG (prevalence < 50%= green, 50 < 70%= brown, 70 < 98%=purple, and 99%< showed with blue color) were illustrated in the bar chart. C: Principal coordinate analysis using Bray-Curtis dissimilarity. The p-value and R squared values based on Adonis2 (with 10,000 permutations) were 9.999e-05, and 0.0848, respectively. The p-value for Betadisper was also significant (*P* = 0.001)

We used vRhyme to construct viral metagenome-assembled genomes (vMAGs), resulting in a final curated set of 167 vMAGs after post-binning quality filtering (Fig. 3 and Supplementary Fig. S5). To determine the prevalence of the recovered vMAGs, we assessed their occurrence across samples based on consistent read-level support and predefined abundance thresholds (Fig. 3A). Out of 167 vMAGs, 11 were identified in more than 70% of the samples. Among these, two genomes whose VPF membership ratios primarily belong to the *Myoviridae*-like lineages, were detected in all 851 samples (Fig. 3B and Supplementary Fig. S5). Importantly, prevalence estimates are based on consistent read-level detection and predefined abundance thresholds rather than complete genome recovery within individual samples.

Next, we further investigated the pattern of viral sequences across the samples, using principal coordinate analysis (PCoA), and clustering for sampling locations was assessed using Adonis2 and Betadisper tests. A significant effect from sampling location was observed not just based on the vMAG sequences but also based on unbinned viral sequences (2381 contigs) (Fig. 3C and Supplementary Fig. S6). Although statistically significant, sampling location explained only a small fraction of total virome variation, and differences in dispersion among groups may partially contribute to the observed signal.

### *Drosophila melanogaster* DNA viruses

During the analysis of virus protein composition, we identified a number of contigs deriving from nudiviruses, which are known to include multiple pathogens of *Drosophila melanogaster* [17]. Although these eukaryotic DNA viruses were not the primary focus of this study, we also present these data as a resource for researchers working on Drosophilidae rather than their bacterial microbiota, as viral presence could be robustly confirmed by per-sample read mapping, and provides biological context for the gut-associated virome.

A blastn search against the NCBI viral genome database showed that the Nudivirus contigs were derived from the gut pathogen Drosophila Kallithea nudivirus (NC_033829) with 99–100% coverage and a 99% identity. By mapping the high-quality short reads to the reference genome, we found that Kallithea virus occurs frequently, with a high overall prevalence of 54.2% (in 461 individuals of 851 samples with genome copy-number threshold ≥ 1% of the fly genome copy-number). The prevalence of Kallithea virus varied among four different sampling locations, ranging from 33.9% to 82.1%. We also detected Drosophila Esparto nudivirus, but its prevalence was extremely low, with only two flies affected.

**Fig. 4:**
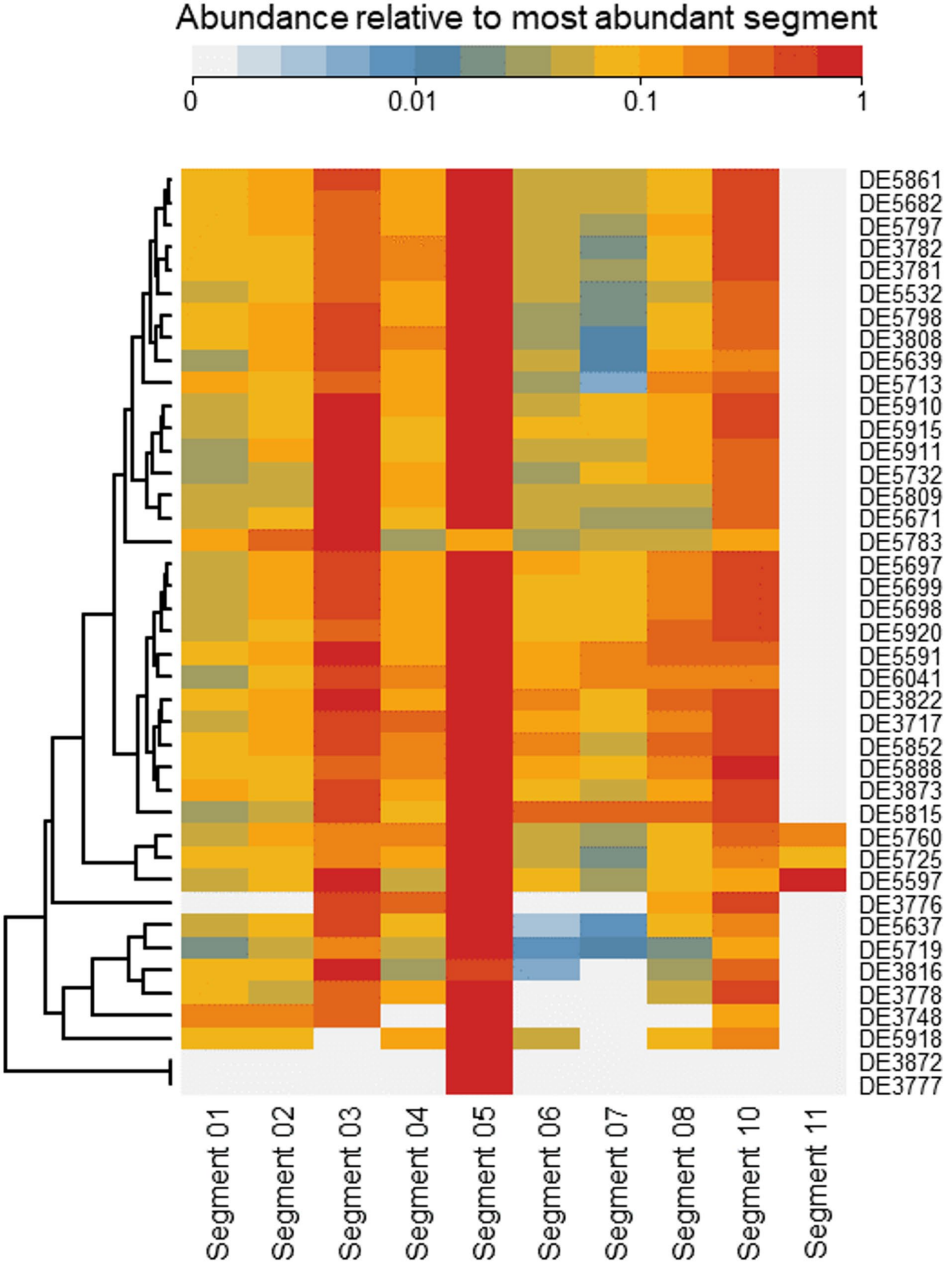
Heatmap demonstrating the relative number of sequencing reads from each of the 10 Vesanto virus segments, for each sample with the minimum threshold of 1%

By mapping reads to all previously reported DNA virus genomes from *Drosophila melanogaster* [15, 17, 21], we identified two further previously known *Drosophila* pathogens, including Drosophila Linvill Road densovirus and Vesanto bidna-like virus. Drosophila Linvill Road densovirus was extremely rare, being detected in only seven individuals, all from sampling location 1, indicating a prevalence of 2% in that location and a total prevalence of 0.8% across the full dataset (95% confidence intervals 0.33%-1.69%). On the other hand, *Drosophila* Vesanto virus, a multi-segmented bidna-like virus, occurred at relatively high prevalence of 20% (95% confidence interval 18–23%; 172 samples out of 851) using a threshold of ≥ 1% (Fig. 4).

Almost all previous sequencing of Vesanto virus has been based on large metagenomic pools, leaving the associations between putative segments uncertain, as many independent infections are represented in each pool [17]. Here, based on single fly sequencing, we did not detect segments 9 or 12, which were also absent from some population pools reported in [17]. Detection of the other segments was variable, with segment 5 being detected in 165 out of 851 samples (Fig. 4A, i.e. nearly all individuals infected with the Vesanto virus), segments 3 and 10 being detectable in 86 and 56 samples respectively and segments 1, 2, 4, 6, 7, and 8 each appearing in more than 35 individuals. Segment 11 was extremely rare, detected in only three samples. Confidence intervals for virus prevalence were calculated assuming a binomial distribution.

Excluding segment 11, all other segments were detectable in those infected individuals that had the highest Vesanto virus read numbers (e.g., samples DE5797 and DE5682, which exhibited almost 2.5 and 1.8 million Vesanto virus reads, respectively). This is consistent with some segments (e.g. segments 9, 11, and 12) being ‘optional’ or ‘satellite’ components and other segments differing in copy-number, with segment 5 being the most readily detectable.

## Discussion

The virome is increasingly acknowledged as an essential part of the microbiome, playing a key role in coevolution with prokaryotic and eukaryotic hosts by both increasing and decreasing host fitness [22]. Here we used a metagenomic approach to elucidate the structure and function of the viral community associated with the gut of wild-caught *Drosophila melanogaster*, with a focus on prokaryotic viruses.

### Phage associated with the *Drosophila* microbiome

Our study found that *D. melanogaster* harbors a diverse community of bacteriophages in their gut (Supplementary Table S2). Lifestyle prediction suggested a predominance of lytic phages, but because lifestyle prediction accuracy depends strongly on contig length and genome completeness, this pattern should be interpreted cautiously and primarily as a descriptive trend among higher-confidence viral contigs rather than as a definitive estimate for the entire catalog. We also observed variations in bacteriophage community composition among sampling locations. Although this effect was statistically significant, sampling location explained only a small fraction of total virome variation, and differences in dispersion among groups may partially contribute to the observed signal. This pattern is consistent with the view that the gut microbiome, together with host-associated and environment-associated factors, is dynamic at fine spatial scales [23, 24]. Given the known dispersal capacity of *Drosophila melanogaster* at regional spatial scales and the ecological similarity among sampling sites, the weak location effect is best interpreted as fine-scale regional variation rather than evidence of strongly isolated fly populations. In line with this, co-assembly by sampling location was used only as a discovery strategy to improve recovery of low-abundance viral sequences, whereas all downstream analyses of viral presence and abundance relied on individual-sample read support. Detection of viral sequences and vMAGs in individual flies was therefore interpreted as read-level support rather than complete genome reconstruction within each sample.

We used two computational approaches for host assignment of viral sequences: the VPF-based host prediction and the CRISPR-spacers similarity. Both methods assigned a relatively low percentage of the phages to a particular host (less than 16% in total). This is consistent with previous findings in honeybee [25] and human [26] virome studies. This limited assignment rate likely reflects incomplete reference databases and the difficulty of assigning hosts to divergent or fragmented viral sequences. Among the contigs with putative host predictions, nearly 70% were linked to bacterial genera commonly associated with the *Drosophila* gut microbiome, including *Lactobacillus*, *Acetobacter*, and *Gluconobacter* [27–29]. This supports the interpretation that at least part of the recovered phage community represents a true fly-associated gut virome. Nevertheless, some viral sequences may also represent food or components of the substrate-associated microbial communities, as *D. melanogaster* feeds on microbes that grow on decaying substrates.

Although bacterial DNA was present in the metagenomic data, detailed bacterial community profiling and MAG-based analyses were beyond the scope of this study. Consequently, host assignments should be interpreted as computational predictions rather than experimentally validated host-phage relationships. Host predictions were therefore not validated by direct co-occurrence with measured bacterial taxa in the same samples and should be considered predictive rather than confirmed associations. However, previous work has characterized the bacterial community composition of *Drosophila melanogaster* across overlapping and nearby European sampling locations, consistently identifying genera such as *Lactobacillus*, *Acetobacter*, and *Gluconobacter* as dominant members of the fly-associated microbiome [30, 31]. The putative host assignments recovered here are therefore broadly consistent with known ecological patterns of *Drosophila*-associated bacteria.

The majority of the viral sequences identified in this study showed limited similarity to previously known phages. A substantial fraction of contigs either remained unclustered or clustered only with other sequences from this dataset, indicating that the *D. melanogaster* gut virome contains viral diversity that is underrepresented in current reference databases. vConTACT2 has been shown to recover clusters consistent with the International Committee on Taxonomy of Viruses (ICTV) classification with high accuracy under benchmark conditions [32], supporting its use here to describe gene-sharing relationships among the recovered viral contigs. Similar patterns have been reported in previous studies on honeybees [25], the human gut [33], and soil phages [34], where only a small fraction of recovered viral sequences matched previously described viruses (less than 12%). However, because bacteriophage taxonomy and reference databases remain incomplete and are undergoing rapid revision, these results should be interpreted as database-derived similarity patterns rather than definitive formal taxonomic assignments. Future reannotation using updated resources, such as INPHARED databases [35], may refine taxonomic placement without altering the broader ecological patterns reported here.

Phages are known to facilitate genetic material transfer in host communities and can affect host phenotypes [36], suggesting that phage-associated proteins may play important roles not just in their life cycles but also in interactions with their host. In this study, the prokaryotic viral-associated proteins that could be assigned to a function were predominantly related to metabolic and DNA processing pathways. Phage-encoded genes involved in DNA replication, repair, recombination, and modification are commonly interpreted as strategies to protect viral genomes from host restriction systems, enhance replication efficiency, and ensure successful propagation during infection, rather than reflecting host-like metabolic functions. The limited information available to categorize many viral proteins further highlights our incomplete understanding of these processes. Similar functional patterns have been reported in honeybee-associated viromes [25], supporting the view that insect gut viromes can include a wide range of genes related to metabolism and genome processing.

Phages can also contribute to the evolution of their hosts [37], particularly in the rewiring of metabolic pathways to meet their requirements [38, 39]. Thus, the presence of pathways related to energy, vitamin, carbohydrate, and amino acid metabolism in the *Drosophila*-gut-associated virome, similar to observations in honeybees [25] and lizard [3], suggests that phages may carry genes with potential roles in microbial metabolic processes rather than solely rely on the host-derived resources. However, these annotations should be interpreted cautiously, because functional inference depends on contig quality, gene prediction, database representation, and viral genomic context. Therefore, the revised analysis focuses mainly on contigs longer than 5 kb, while full-catalog annotations are retained as a supplementary resource.

In the context of phage-host interaction, the presence of accessory metabolic genes (AMGs) can determine the success of phage proliferation [5]. In this study, putative AMGs detected among longer *D. melanogaster* gut phage contigs were mainly associated with amino acid metabolism, cofactor and vitamin metabolism, and folding, sorting and degradation. The frequent detection of genes such as dcm, which encodes DNA cytosine methyltransferase, and queE, involved in 7-cyano-7-deazaguanine biosynthesis, is consistent with functions that may help phages evade host defense systems or modify nucleic acids [40–42]. These findings are broadly consistent with previous studies on human and urban environments [2]. We speculate that these AMG functions may reflect not only the needs of the phages, but also features of their microbial host and environmental context [34]. Such symbiotic interactions may contribute to microbial metabolic processes within the gut ecosystem and could potentially influence host-microbiome interactions [43, 44]. However, these AMG annotations represent computational predictions and should not be interpreted as experimentally validated metabolic functions. The lower number of AMGs retained among contigs longer than 5 kb and classified by CheckV as at least medium quality further highlights the importance of contig quality and genomic context for AMG interpretation.

The KEGG pathway summary also indicated that longer phage contigs encoded genes associated with a range of metabolic and genetic-information-processing categories, including carbohydrate, energy, lipid, nucleotide, and amino acid metabolism, as well as replication, repair, transcription, translation, and protein processing. Pathways related to biofilm formation, and bacterial secretion system may suggest potential interactions between phages and bacterial hosts, but these results remain descriptive and require experimental validation. Similar observations in honeybee viromes have suggested that phage-associated functions may be linked to bacterial processes within the gut ecosystem [25]. Moreover, the detection of xenobiotic degradation and a limited number of terpenoids and polyketides-related functions may indicate additional layers of phage-bacterial interaction, although these annotations should be interpreted cautiously. Overall, rather than demonstrating direct viral control of bacterial metabolism, the functional and AMG results provide hypotheses for future studies on how phages may interact with bacterial members of the Drosophila gut microbiome and potentially contribute to host-microbiome interactions.

### DNA viruses infecting *Drosophila*

We found only four DNA viruses that are likely to infect the fly rather than its microbiome consistent with previous claims that DNA viruses of *Drosophila melanogaster* are relatively rare [17]. These included Drosophila Kallithea nudivirus, Drosophila Esparto nudivirus, Drosophila Linvill Road densovirus, and Drosophila Vesanto virus. Although our study primarily focuses on bacteriophages, detection of known Drosophila DNA viruses provides an internal biological validation of the sequencing and mapping strategy and may be of broader relevance to the Drosophila research community.

Drosophila Kallithea nudivirus is a large dsDNA virus that is closely related to a number of other known *Drosophila* pathogens and has been shown to be pathogenic in adult flies [15, 17, 40, 45]. We found that *Drosophila* Kallithea nudivirus had high prevalence across our four sampling sites, with infection rates exceeding 50% of individuals. This is consistent with a previous metagenomic study of DNA viruses infecting European *D. melanogaster*, which reported that *Drosophila* Kallithea nudivirus infects more than half of populations [17].

Drosophila Vesanto virus was the second most common virus of *Drosophila*, being detected in more than 20% of the individuals. Here, using data from single flies, we detected only 10 out of 12 previously described segments of Drosophila Vesanto virus, and we observed distinct variation in the detection of the segments, with segment 11 appearing extremely rare. Based on large pool-sequencing datasets [17], three hypotheses have previously been proposed to explain this pattern: that there are 12 segments with extreme variation in copy number; that there is a reassorting community of segments in which some are optional or satellites; or that Vesanto is not a single virus, and multiple viruses appeared in each of the pools presented there—perhaps infecting a component of the cellular microbiome. The very strong association we see here between the majority of the segments (and the absence of shared eukaryotic microbiome from some affected individuals) strongly suggests that these are indeed segments of the same virus, as argued by [17] on the basis of a single infected lab line. The complete absence of two segments from all affected flies further confirms that some segments are indeed optional or satellite. However, the lack of negative correlations among most segments, along with the generally consistent pattern of copy-number variation, suggests that in general (apparent) absences are likely driven by the relatively low titer of some segments, rather than segments being optional or homologous being able to substitute for each other. Such variation in genome copy number among infections may hint that Vesanto virus has a multipartite lifestyle [46, 47].

## Methods

### Sample collection and metagenomic sequencing

A total of 851 male *Drosophila melanogaster* individuals were collected from four sampling locations in Germany over a two-month period between late August and early October 2019. The four sampling locations were selected based on logistical feasibility for repeated wild sampling within Baden-Württemberg, rather than to represent predefined environmental or habitat categories, and were separated by short geographic distances within the Freiburg region (for more details see Supplementary Fig. S7). For sample collection from all sites, we used traps baited with a mixture of apple and cherry, following a standardized protocol [21]. The number of flies collected per location varied due to differences in trap yield. The flies were anesthetized on a fly pad using CO_2_ and subsequently transferred into empty Eppendorf tubes. The Eppendorf tubes were then dipped into liquid nitrogen to freeze the samples. Following this, all fly samples were dissected to extract the gut, and then the individual gut samples were stored in PBS at -80 °C until DNA extraction.

We extracted DNA from each gut sample individually following the DNA extraction protocol described in detail in [21] with slight modification. Briefly, the samples were homogenized using a bead beater (Qiagen Tissue Lyzer II), with the addition of lysozyme to improve the efficiency of nucleic acid extraction. Protein was digested with Proteinase K, and RNA was removed using RNAse. We precipitated DNA using a combination of Phenol-Chloroform-Isoamyl alcohol and after washing with alcohol the DNA was resuspended in nuclease-free water. The NEBNext^®^ Multiplex Oligos with unique dual index primer pairs for Illumina was used for library preparation. Then samples were sequenced on the Novaseq 6000 platform with 150 bp paired-end sequencing strategy at the Life & Brain research centre (University Hospital Bonn).

### Metagenome assembly

We used Cutadapt [48] and FastQC [49] implemented in TrimGalore v0.6.6 [48] to trim adapters and control the quality of the raw reads. Then, to remove human and host sequences, we mapped the genomic DNA of the data from each of the 851 samples to the host *D. melanogaster* and the human genome (hg38), using bbmap [50]. After removing host-derived reads, a median of 5.1 million non-fly paired-end read pairs per sample (range: 23,436–73,879,545) was retained for downstream analyses, totaling 7.7 billion non-fly paired reads across all samples (Supplementary Table S7). Finally, we assembled the remaining paired-end reads using MEGAHIT [51]. To improve the assembly of low-abundance components of the microbiome, we co-assembled reads from multiple individuals, pooling by sampling location, for a total of four co-assemblies. Because flies collected at the same site are not guaranteed to be closely related, these co-assemblies were used strictly as a contig discovery step. Viral sequence presence and abundance were subsequently assessed at the individual-sample level by mapping filtered reads back to the recovered contigs, interpreting detection as read-level support rather than genome reconstruction, and applying downstream quality and completeness filtering to minimize potential chimeric artifacts.

### Recovering high-quality viruses and binning of viral genomes

We predicted viral contigs among metagenome contigs using VIBRANT v1.2.1 [18] with the default parameters. VIBRANT uses machine learning and a protein similarity approach to accurately recover bacteriophages [52]. In outline, VIBRANT uses the contigs predicted by Prodigal v2.6.3 [53] and annotates them with the Kyoto Encyclopedia of Genes and Genomes (KEGG) KoFam [54], Pfam [55], and virus orthologous group (VOG) [56] databases using HMMER v3.3.2 [57]. At the same time, we also inferred auxiliary metabolic genes (AMG) using VIBRANT. VIBRANT identifies AMGs only when metabolic genes are embedded within contigs classified as viral based on viral hallmark gene context and machine-learning classification; therefore, AMGs reported here originate from viral contigs rather than from standalone bacterial sequences. VIBRANT employs neural networks of protein signatures, and a novel v-score metric to overcome conventional limitations, enabling comprehensive identification of lytic viral genomes, thereby capable of providing estimates of genome quality and differentiation between lytic and lysogenic viruses.

The completeness and quality of all phage contigs were assessed using CheckV v1.4 [58] with the parameters “end_to_end” and removing all contigs shorter than 3 kb. To ensure the quality of retained phage contigs, we ran VirSorter v2.2.3 [19] and DeepVirfinder (v. 1.0) [20] against the same assembled data. We considered the phage contigs recovered by VIBRANT as supported bacteriophage predictions if VirSorter2 (groups include-dsDNAphage, ssDNA viruses, NCLDV-Nucleocytoviricota) and DeepVirfinder scores were ≥ 0.5.

DeepVirFinder has especially been advocated for the identification of short (1 < kbp) phage genome fragments [59]. The dsDNA, ssDNA and NCLDV, respectively, represent double-strand and single-strand DNA viruses and nucleo-cytoplasmic large DNA viruses. However, to reduce the chance of false positives given the sensitive cut-off point for the length of the VIBRANT and VirSorter2 fragment of 3 kbp, the short contigs < 3 kbp) were also retained if they had been recovered by all three pieces of software and had VirSorter2 and DeepVirfinder quality scores ≥ 0.9. Contigs with provirus contamination were also retained if they exhibited a clear shift from the host genome and met the aforementioned criteria, after extraction of the proviral region.

All identified contigs from the four co-assemblies were pooled and dereplicated (–method longest) using vRhyme v.1.1.0 [60]. The quality of the final nonredundant phage sequences has been assessed using CheckV v1.4 [58]. We also used Cenote-Taker 2 v2.1.5 with “-am True” [8] to further assess and annotate the nonredundant recovered sequences. For downstream analyses, we distinguished between a comprehensive viral catalog generated for discovery purposes and a high-confidence subset used for ecological, taxonomic, lifestyle, and functional interpretation. The high-confidence subset consisted of viral contigs supported by at least two independent viral identification tools (VIBRANT, VirSorter2, and/or DeepVirFinder), and either exceeding 5 kb in length or classified as medium or high quality according to CheckV, in accordance with MIUViG recommendations. Shorter or lower quality contigs were retained in the full catalog for completeness but were not used to support key biological conclusions.

Finally, we used the nonredundant viral contigs binning strategy to group scaffolds into putative genomes using vRhyme [60] with the default parameters. As part of bin construction, vRhyme checks for protein redundancy and this metric can be used for post-binning filtration (Redundant protein < 1 unlikely to be contaminated: 2 < 5 may not be contaminated; >6 are more likely to be contaminated) [60].

To perform a quantitative analysis of the obtained phage contigs, we used metaWRAP with “quant_bins” module to map the filtered non-fly paired-end reads from each individual sample against the nonredundant viral contigs and vMAGs. Then the average abundance of each (viral metagenome-assembled genome) vMAG for each of the 851 samples was calculated using Salmon [61], reporting quantities as transcripts per million (TPM) [62] and taking the length-weighted average of the contig abundances. As expected for non-viral-enriched single-fly gut metagenomes, viral reads constituted only a small fraction of the retained non-fly reads, consistent with previous insect gut virome studies. All viral contigs discussed in the Results, including eukaryotic DNA viruses, were confirmed by read mapping at the individual-sample level rather than inferred solely from co-assembly.

We performed principal coordinate analysis (PCoA) and visualized the Bray–Curtis distances of relative viral abundances at each sampling location using the vegan package (v2.5-7) in R v4.2.0 [63]. Then the observed variation was further tested using Adonis2, also Betadisper test in the vegan package [64], to gain a better understanding of the underlying patterns and structure in the data set as well as validate the findings obtained through PCoA. Adonis2 helps determine if the observed groupings in PCoA are statistically significant, while Betadisper provides insights into the within-group variability and helps identify if the groups identified by PCoA have significantly different spread or variability. We also made a heatmap to visualize the differences between phage composition of different localities using Seaborn clustermap v 0.12.2 [65]. P-values from PERMANOVA analyses were interpreted descriptively, and no correction for multiple testing was applied.

### Protein clustering and taxonomy of viral contigs

We evaluated viral contig similarity and constructed a viral protein clustering network with vConTACT2 0.11.3 [32] using the viral RefSeq v211 database. vConTACT2 infers gene-sharing relationships among viral genomes, and resulting clusters are interpreted as network-based taxonomic groupings rather than formal ICTV classifications. The vConTACT2 method uses gene-sharing networks to assign the taxonomy of viruses based on their sequence. The viral sequences are then classified as ‘clustered’ (high-confidence clustering), ‘overlap’ (sharing overlap gene content), ‘outlier ‘(weakly associated with a cluster) or ‘singleton’ (few or no shared gene content) [32]. The network was visualised using Cytoscape v3.9.1 [66]. We performed host assignment and taxonomic annotation at the family and genus level with VPF-Class [67] using VPF classified files from 17 August 2022 to build the reference database. For preliminary taxonomic annotation, we considered a VPF membership ratio cutoff of 0.2 and 0.3 at the family and genus level, respectively.

### Host assignment of recovered bacteriophage contigs

To assign viral genomic sequences to their specific host, we applied two strategies. First, we used VPF-class tool, which takes advantage of the Viral Protein Families (VPFs) and creates a protein-based database from the IMG/VR database. The VPF-class tools then conducts a protein family search for each query genome and concludes the host prediction based on the distribution of these VPFs in reference phage genomes, first at the domain and then at the host family and genus levels [67]. In the second approach, we used CRISPR spacer-based bacterial host predictions strategy [68]. The CRISPR spacer-based approach uses information from CRISPR–Cas, which works as adaptive immune systems, and has access to a database of more than 11 million spacers [68]. We therefore searched the spacer database with our viral sequences using blastn and predicted bacterial hosts at the genus level on the basis of sequence similarity.

### DNA viruses of *Drosophila melanogaster*

Initial taxonomic assignment of the recovered contigs suggested that at least one nudivirus was present. We therefore used blastn to test whether this was a previously known viral pathogen of *Drosophila*. To detect the presence of other previously known DNA viruses of Drosophila, we mapped the high-quality short reads to the reference genomes of Drosophila Kallithea nudivirus, Drosophila Viltain densovirus, Drosophila Linvill Road densovirus, and Drosophila Vesanto bidna-like virus to quantify the amount of each virus in each fly [15, 17, 21]. We set a threshold of 1 per cent of the fly genome copy number to define infection status, and using these data, we were able to estimate the prevalence of these fly pathogens in our 851 samples.

## Supplementary Information

Below is the link to the electronic supplementary material.

Supplementary Material 1

Supplementary Material 2

## Data Availability

The sequencing data generated and supporting the findings of this study are deposited in the NCBI Sequence Read Archive (SRA) under BioProject accession number PRJNA1097789 [https://www.ncbi.nlm.nih.gov/bioproject/PRJNA1097789]. Scripts for the bioinformatics processing are publicly available at https://github.com/Minamehr/Manuscripts/tree/main/Virus-Drosophila-melanogaster.

## Abbreviations

AMGs: Auxiliary metabolic genes
MVCs: Metagenomic viral contigs
vMAGs: Viral metagenome-assembled genomes
VPF: Viral protein families
DTRs: Direct terminal repeats
ITRs: Inverted terminal repeats
PCoA: Principal coordinate analysis
