# Supplementary material for: A diverse gut virome in natural populations of Drosophila melanogaster
Source: Anim Microbiome. 2026 Jun 4;8:80. doi: 10.1186/s42523-026-00585-2 (PMC13267423; doi:10.1186/s42523-026-00585-2)
Supplement: Supplementary file 1 — Supplementary Material 1 [file 42523_2026_585_MOESM1_ESM.pdf]

Supplementary Figures.

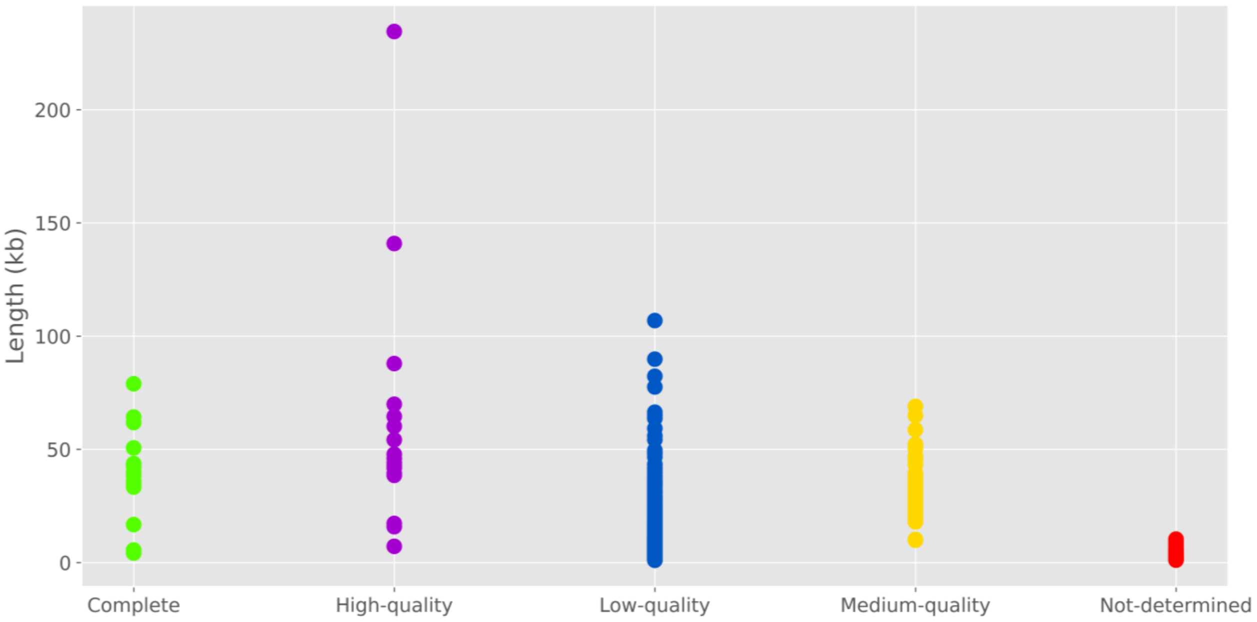

Figure S1. Estimated viral genome qualities as a function of contigs lengths.

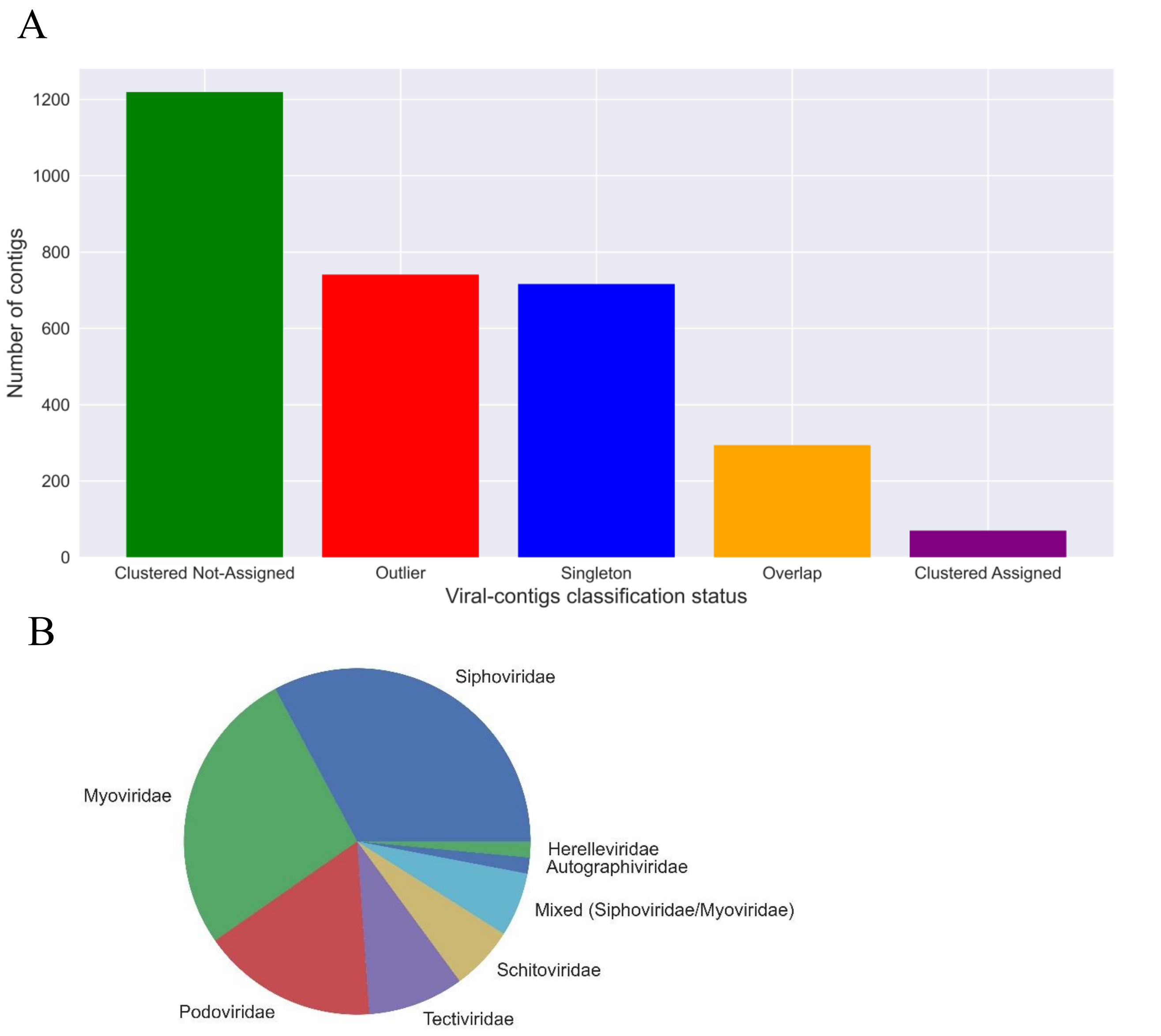

Figure S2. An overview of the recovered viral contigs classification in association with previously described viruses from Refseq database using vConTACT2. A: Representing classification status, “Clustered Assigned” indicate recovered contigs falling into clusters contained reference sequences; “Clustered Not-Assigned” means recovered contigs clustered without reference sequences. B: Referring to “Assigned Viral contigs” at the family level.

A

B

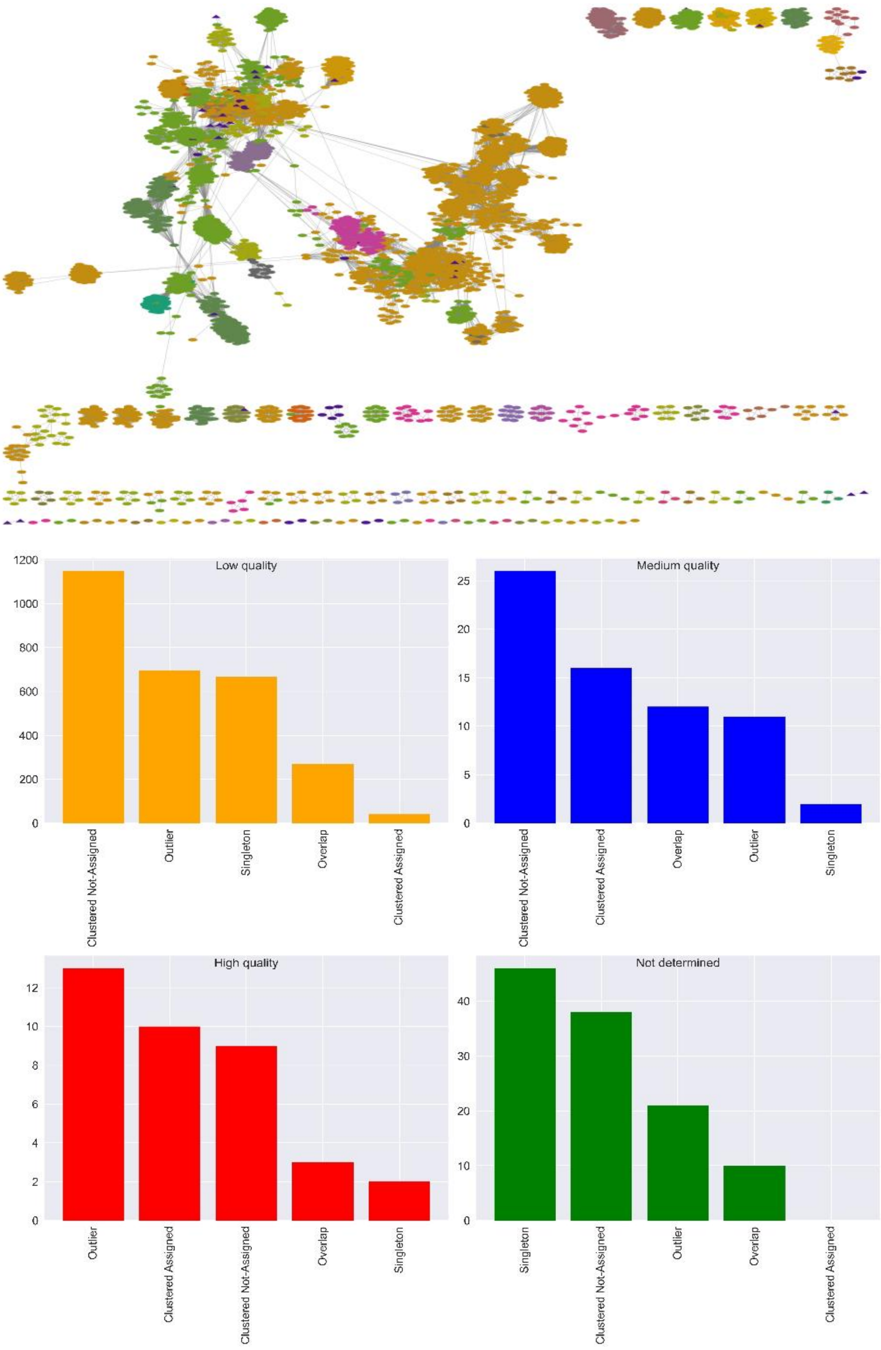

Figure S3. Protein similarity and network analysis constructed with vContact2 and Cytoscape. A: Protein clustering network of the recovered High-quality viral sequences from *D. melanogaster* (purple) compared to previously described viruses from Refseq (other colors). B, C, D and E respectively indicate the classification status of the high, medium and low quality and not-determined quality viral sequences.

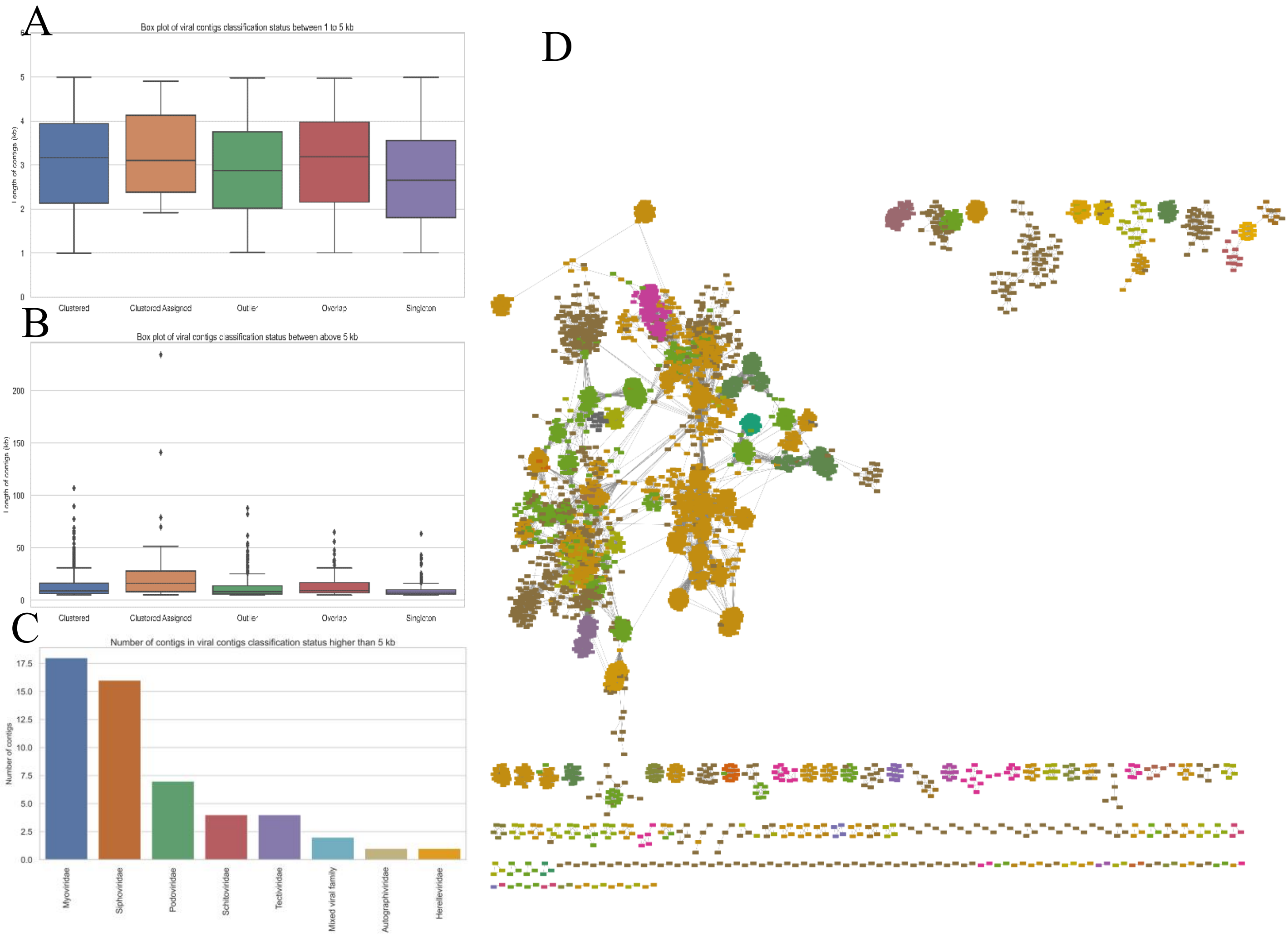

Figure S4. An overview of the recovered viral contigs classification status in association with previously described viruses. A: Boxplot represent the classification status of the short recovered viral contigs (<5 kb) using vConTACT2. B: Boxplot represent the classification status of the large recovered viral contigs (>5 kb) using vConTACT2. C: Number of clusters that includes both confidently clustered large contigs and viral reference sequences. D: The network clustering presenting the retrieved clustered putative large (>5 kb) prokaryotic viruses (dark brown) and the viral family of Refseq (other colors).

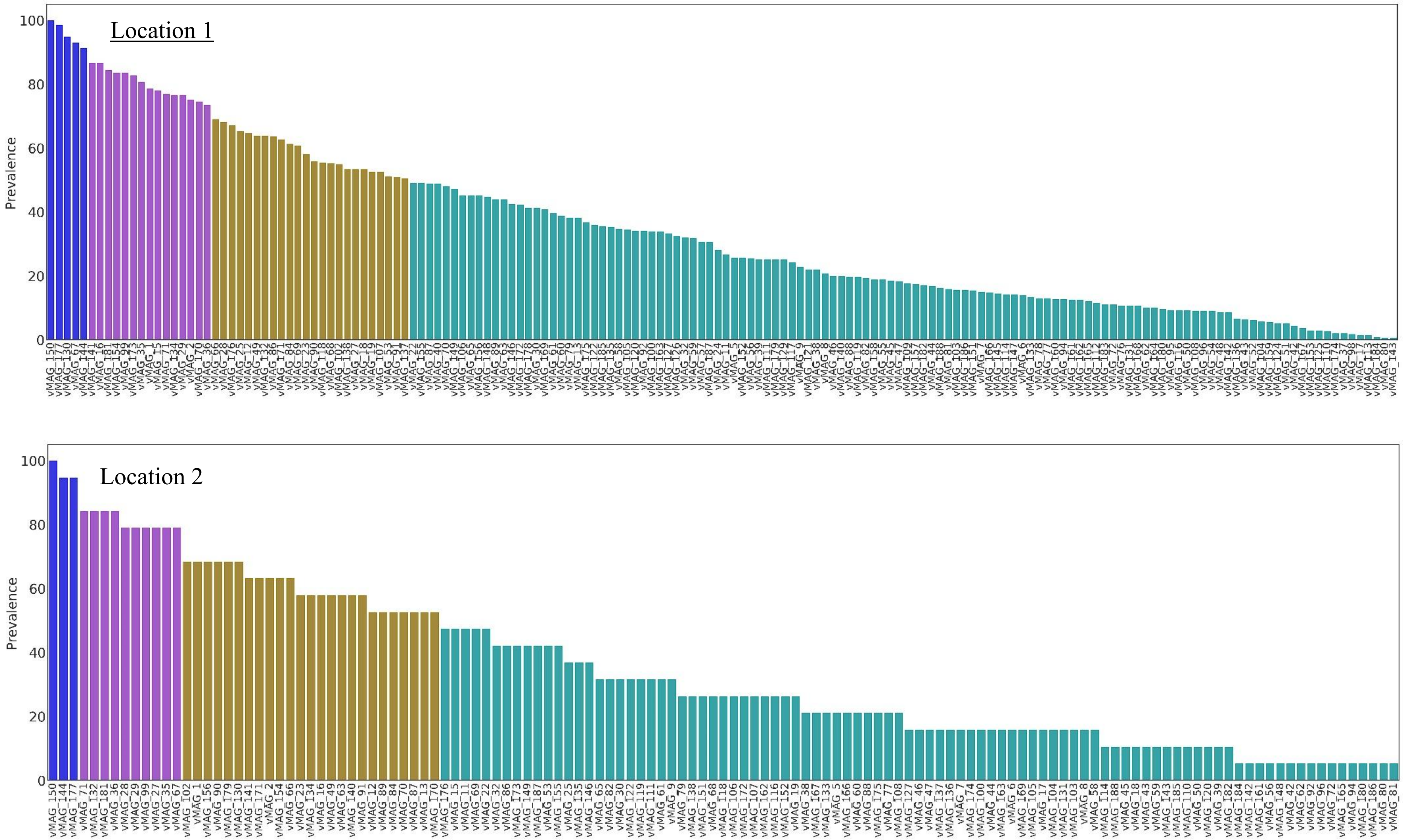

Figure S5_A. The prevalence of each vMAGs (prevalence <50%= green, 50<70%= brown, 70<98%=purple, and 99%< showed with blue color) in each sampling location were shown with the bar charts separetly.

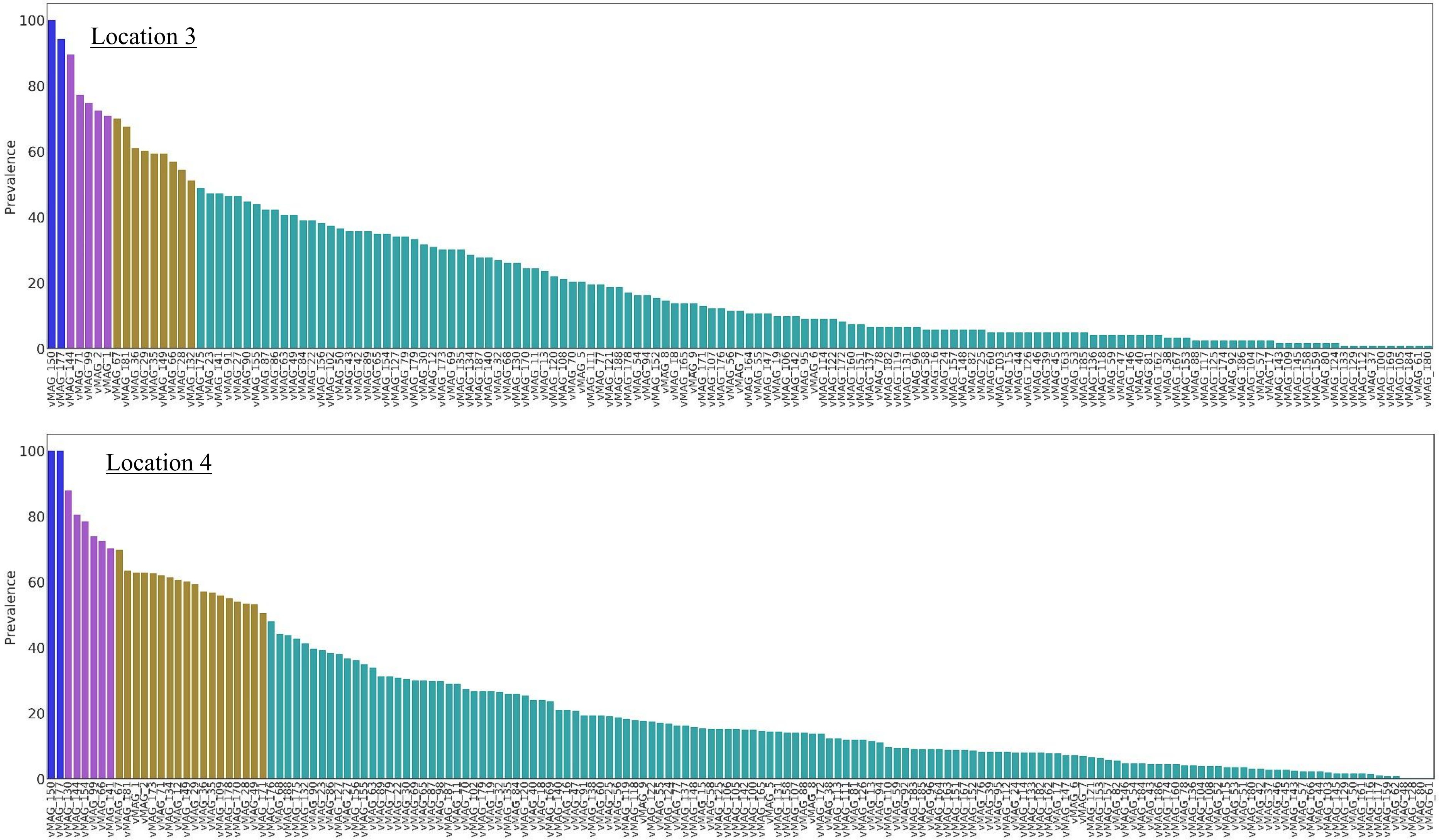

Figure S5_B. The prevalence of each vMAGs (prevalence <50%= green, 50<70%= brown, 70<98%=purple, and 99%< showed with blue color) in each sampling location were shown with the bar charts separetly

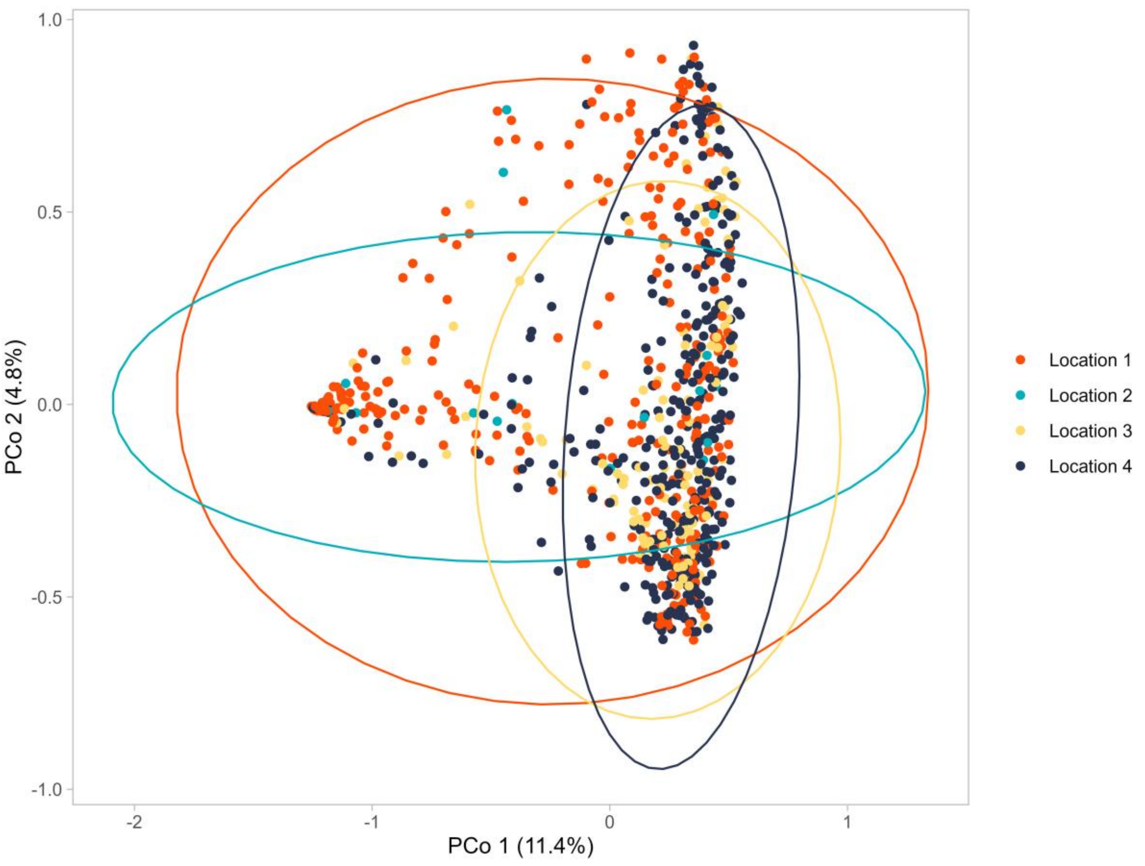

Figure S6. Principal component analysis using Bray–Curtis dissimilarity and based on unbinned viral sequences. P-values of Betadisper was P = 0 .001, also P-values and R squared values from the Adonis2 (with 10,000 permiutation) were P= 9.999e-05 and R= 0.0752, respectively.

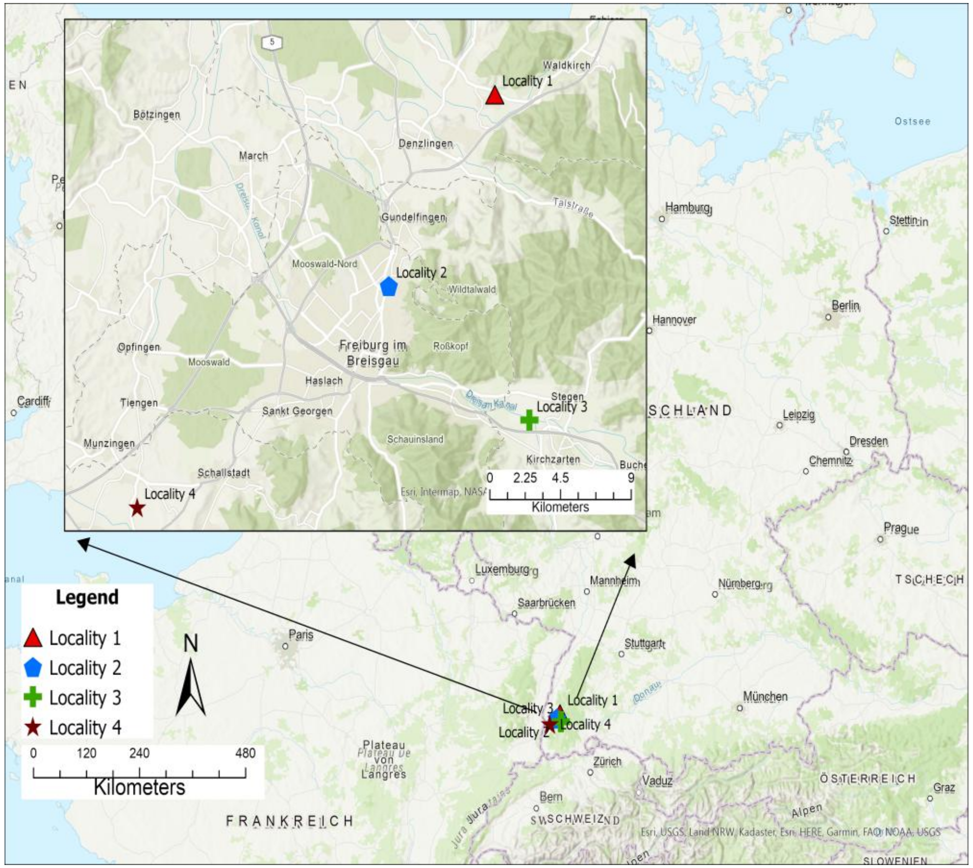

Figure S7. A map representing four sampling locations of *Drosophila melanogaster*. All four sampling locations are within the Freiburg region and separated by short geographic distances (maximum pairwise distance ~20 km). We used a mixture of apples and cherry as bait and placed them in multiple 1.5-liter PET bottles at each sampling site for several days. The final dataset included gut metagenomes from 347, 19, 123, and 362 individual flies from sampling locations 1, 2, 3, and 4, respectively.
